# Status of Research Funding in Nepal: A Scoping Review

**DOI:** 10.31729/jnma.8640

**Published:** 2024-07-31

**Authors:** Mohan Raj Sharma, Sugat Ratna Tuladhar, Abhishek Adhikari, Akriti Khadga, Shreejana Singh, Namita Ghimire

**Affiliations:** 1 Department of Neurosurgery, Tribhuvan University Teaching Hospital, Maharjgunj, Nepal; 2 Research Department, Institute of Medicine, Tribhuvan University, Maharjgunj, Nepal; 3 Nepal Health Research Council, Ramshahpath, Kathmandu, Nepal

**Keywords:** *funding*, *grants*, *low and middle-income countries*, *research*, *scoping review*

## Abstract

With the rapid advancement of health delivery, there has been a renewed interest in conducting research among healthcare professionals in Nepal. However, concern is there regarding availability of funds and mechanisms of awarding. The purpose of this scoping review is to map the available evidence regarding the evolution and current status of health research funding in Nepal and to highlight gaps and areas for future research. We searched three databases for empirical papers and several gray literature. Our search, conducted between March and April 2024 yielded 76 documents of which 30 that met the selection criteria were included in the scoping review. Almost all studies identified lack of funding as a deterrent to research. We found a paucity of research focusing on the role of researchers in funding decision-making. Our findings revealed that there are 12 national and four international organizations providing funds for research. University Grant Commission is the largest funder from Nepal whereas the Research Council of Norway is the biggest international funder. There were certain barriers and facilitators for obtaining funds identified by this scoping review. Further efforts are needed to increase the amount and availability of funds in Nepal to enable high-quality research.

## INTRODUCTION

The mechanisms for funding healthcare research are generally clear in many countries though they are less granular in developing countries.^1-4^ Research performed in Nepal has been increasing over the last 10 years.^5^ Various national organizations such as the University Grant Commission (UGC), universities, and institutions provide funds.^6,7^ The major international funding agencies are the World Health Organization (WHO), the Research Council of Norway (RCN), and the National Institute of Health (NIH).

Literature is scarce regarding the funding opportunities for health researchers in Nepal. The primary objective of this review is to describe the status of health research funding in Nepal. Specifically, we describe the major funding organizations, the mechanisms for funding decisions, the amount given and the ways health researchers contribute to existing funding policies. Also, the possible barriers and facilitators for acquiring health research funding will be explored. This study was registered in Open Science Forum registries.^8^

## METHODS

This review was performed following the recommendation by Arksey and O'Malley's framework comprising a six-stage process: (a) identifying research questions; (b) identifying studies; (c) study selection; (d) data charting; (e) collating, summarizing and reporting results; and (f) an optional stage of consultation exercise.^9^ The report is based on the Preferred Reporting Items for Systematic Reviews and Meta-Analyses Extension for Scoping Reviews (PRISMA-ScR) guidelines.^10^

## RESEARCH QUESTIONS

1. Which organizations are significant sources of funding?
2. What is the funding amount offered?
3. How is the funding decision made?
4. How do health researchers in Nepal contribute to the existing funding policy?
5. What are the barriers/facilitators for acquiring health research funds in Nepal?

## IDENTIFYING STUDIES

Potentially relevant documents were identified based on their titles and abstracts by a literature search in PubMed, Google Scholar, and NepJOL databases using the following search terms (alone or in combination with): "Funding", "Health research", "Nepal" and "Health Research Policy". Gray literature (official websites, position statements, and white papers of governmental and non-governmental organizations, and institutions known to fund research) was also included. The following were our selection criteria:

## INCLUSION CRITERIA

1. Papers related to research funding in healthcare in Nepal,
2. Papers published after 1991 [the year of establishment of the Nepal Health Research Council (NHRC) and UGC], the major funders for research in Nepal,
3. Papers written in English, Nepali, or if English or Nepali translation is available.

## EXCLUSION CRITERIA

1. Papers on research funding for animal studies,
2. Retracted publications,
3. Publications where neither abstract nor full text was available

The final search results were exported to Rayyan software (free version) for screening of the literature.^11^

## STUDY SELECTION

Abhishek Adhikari and Sugat Ratna Tuladhar independently screened the papers. A calibration exercise was performed with 10 random papers from the screened documents. The results were discussed and any disagreements on study selection were resolved by consensus and/or discussion with Mohan Raj Sharma. In the first round 80% agreement was achieved. The disagreements on study selection were again reviewed in the team. In the second round, there was 100% calibration between reviewers. Thus, a final data extraction form (Box 1) was subsequently developed which was also similarly calibrated with 10 random papers. Data extraction was performed by Akriti Khadga and Shreejana Singh when the agreement was >90% between them.

**Box 1:** Data extraction form.

1.	Name of the funding organization
2.	Categories of health research (intramural vs extramural)
3.	Amount offered
4.	Funding decision mechanism
5.	Top funders
6.	Top fund-receiving institutes
7.	Role of health researchers in funding mechanism
8.	Barriers/facilitators for acquiring health research funds in Nepal
9.	Trend of health research funding in Nepal

## DATA CHARTING, COLLATION AND ANALYSIS

A coding framework on topics was made for analyses. The coding framework was developed for the points in the data extraction form. For those with yes/no type of questions 0 and 1 was used whereas for multiple response 0 to the number based on the number of responses was performed. A descriptive analysis of the data was undertaken.

## RESULTS

**Figure 1:**
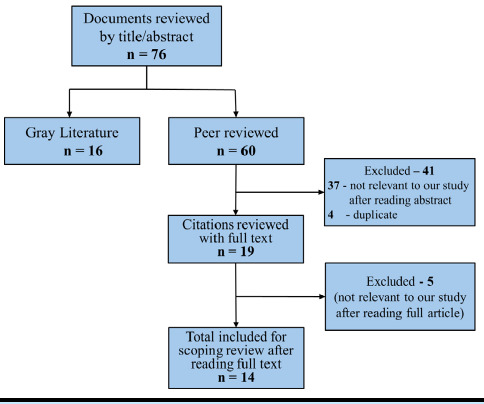
PRISMA diagram depicting the document selection process for the scoping review.

The process of selection of the literature is depicted in (Figure 1).

We screened 76 documents based on their titles and abstracts and we included 14 peer-reviewed articles (Table 1) and 16 documents from the gray literature (Table 2) for our study based on selection criteria.

### Article Profile of Peer-reviewed Literature

Of the reviewed journals, 10 were PubMed indexed whereas the remaining four were NepJol indexed. Four articles discussed the funding organizations,^2,12-14^ whereas only one article described the number of funds provided by different organizations.^12^ None dealt with the funding mechanisms and contribution of researchers to funding policy. Twelve articles discussed the barriers to research and publication (all except articles 6, and 9 in Table 1).

**Table 1:** Authors, title, year of publication and main results of the retrieved peer-reviewed articles for scoping review.

Serial no.	Article	Authors	Journal and year of publication	Primary focus
1.	Health research in medical colleges	Acharya, GP^15^	Kathmandu Univ Med J., 2004	Discusses the barriers to conduct research and suggests ways to promote health research in medical colleges.
2.	Initiating and strengthening medical student research: time to take up the gauntlet	Shankar, PR, Chandrasekhar, TS, Mishra, P, et al^16^	Kathmandu Univ Med J., 2006	Discusses the ways medical students can carry out more research.
3.	Challenges and opportunities of public health research in Nepal	Shrestha, S^17^	Kathmandu Univ Med J., 2014	Discusses the importance and challenges of public health research in Nepal.
4.	Towards diaspora-driven research capacity strengthening in low- and middle-income countries: results from India and Nepal	Varadaraj, V, Ranjit, A, Nwadiuko, JC, et al^18^	Int Health. 2019	Recommends involving diaspora health workers in research capacity strengthening in LMICs.
5.	Is research in peril in Nepal? Publication trend and research quality from projects funded by the University Grants Commission-Nepal	Paudel, P, Giri K, Dhakal B, et al^19^	Account Res., 2020	Discusses the trends in funding by the University Grant Commission Nepal.
6.	Health research capacity in Nepal: Analysis of the trend and the role of local researchers	Kharel, M, Pokharel, A, Sapkota, K et al^12^	Trop Doct., 2021	Analyzes the trend of health research in Nepal.
7.	Gender inequality in the global mental health research workforce: a research authorship scoping review and qualitative study in Nepal	Gurung, D, Sangraula, M, Subba, P, et al^3^	BMJ Glob Health., 2021	Looks at gender equality in mental health research in Nepal.
8.	Implementation of National Science Technology and Innovation Policy 2019: assessment of challenges in Government Organizations of Nepal	Poudel, RC^2^	Nepal Journal of Science and Technology, 2021	Discusses the challenges researchers face in Nepal.
9.	Priorities for cancer research in low- and middle-income countries: a global perspective	Pramesh, CS, Badwe, RA, Bhoo-Pathy, N, et al^1^	Nat Med., 2022	Discusses the shortcomings in research in LMICs and ways to overcome them.
10.	Perceived barriers to publication in scholarly journals by faculty from Maharajgunj Medical Campus	Dahal, S, Shrestha, GS, Singh, S, et al^13^	J Nepal Health Res Counc., 2022	Highlights barriers to research and publications in academic settings.
11.	Research capacity for prevention and control of non-communicable diseases and their risk factors in Nepal: Findings of a needs assessment study	Oli, N, Pradhan, PMS, Sagtani, R A, et al^20^	Kathmandu Univ Med J., 2022	Discusses the disparity between a high burden of non-communicable diseases and low research output.
12.	Article processing charges - A challenge for researchers in Nepal	Shankar, PR and Jha, N^21^	Journal of Chitwan Medical College, 2022	Shows that publications in Nepal have grown significantly over the past two decades.
13.	The role of NHRC in regulating health research ethics in Nepal: A narrative review	Adhikari, A, Aryal, B, Devkota, S, et al^6^	Journal of Health Promotion, 2023	Reviews the role of the Nepal Health Research Council in promoting research.
14.	Neurosurgeons as researchers in developing countries	Sharma, MR^4^	Annapurna journal of Health Science 2023	Discusses how high-income country research is not generalizable in LMICs due to different patient needs and resource availability.

LMICs = Low and middle-income countries

## FUNDING ORGANIZATIONS

Among funding organizations, three were government bodies (NHRC, UGC, and Nepal Academy of Science and Technology (NAST)), six were either research directorates of universities (Tribhuvan University (TU), Kathmandu University (KU), Pokhara University (PU), Madan Bhandari Academy of Health Sciences (MBAHS)) or their respective campuses or hospitals, one was a professional society (Nepal Pediatric Society (NEPAS)), two were nongovernmental organizations and remaining four were international organizations (Table 2).

**Table 2:** List of organizations providing research funding in Nepal.

SN	Name of the Organization/Institute
**National**	
1.	University Grant Commission Nepal^22^
2.	Nepal Health Research Council^23^
3.	Nepal Academy of Science and Technology^24^
4.	Tribhuvan University Research Directorate^25^
5.	Directorate of Research, development and Innovation, Kathmandu University^26^
6.	Dhulikhel Hospital, Kathmandu University Hospital Research and Development Division^27^
7.	University Research Center, Pokhara University^28^
8.	Research Department, Institute of Medicine, Tribhuvan University^29^ Maharajgunj Medical Campus, Institute of Medicine, Tribhuvan University^30^
9.	Nepal Pediatric Society^31^
10.	Birat Nepal Medical Trust^32^
11.	Madan Bhandari Academy of Health Sciences (MBAHS)^33^
12.	Institute for implementation science and health^34^
**International**	
13.	National Institute for Health^35^
14.	Research Council of Norway^36^
15.	World Health Organization^37^
16.	John Hopkins University^38^

## UNIVERSITY GRANTS COMMISSION

The commission funds various programs in Nepal's higher education institutions as described below:^39^

a. Small Research Development and Innovation Grants. Top institutes receiving this funding in the year 2022/23 include Maharajgunj Medical Campus (MMC) and Nepal Medical College.
b. M. Phil. Fellowship
c. Ph.D. Fellowship. KU was a notable recipient of this fellowship in the year 2022/23.
d. Collaborative Research Grants. KIST Medical College and Teaching Hospital was the key recipient of this grant in 2022/23.
e. Faculty Research Grants. Top receiving institutes include Patan Academy of Health Sciences and Manmohan Memorial Institute of Health Sciences in 2022/23.
f. Support for publication of articles in Scimago-ranked journals.
g. Research infrastructure development support
h. Research promotion grants

The overall budget spent by UGC to fund research activities in the year 2022/2023 was 145000000 Nepalese rupees (NPR) (approximately USD 1100000.00).^22,39^

UGC requires a midterm presentation of the ongoing research and submission of the final draft and publication in a UGC recognized journal before releasing the final 30 % of the fund.^22^

### Nepal Health Research Council

Various programs from NHRC support undergraduate to PhD scholars as well as freelance researchers.^40^ NHRC stratifies grants according to Nepal's political division into seven provinces. The number of awards varies every year, reflecting the dynamic nature of the research landscape and the availability of funds. Nevertheless, NHRC consistently awards more than 10,000,000.00 NPR each year.^40^

### National Academy of Science and Technology

There is limited information on the funding mechanism of this organization. The criteria emphasize higher education qualifications, specialized knowledge, publication records, and being a government employee. Young Scientist research grant allocates 16% to multidisciplinary research, 26% to institutional research, and 58% to individual/group research, with 15% of total funds dedicated to medicine and health sciences.^24,41^

**Tribhuvan University Research Directorate**

The Directorate supports faculty members in their research. The grants are categorized into:

1. Emerging Faculty Research Grant (EFRG): One million NPR
2. Major Research Grant (MRG): Two million NPR
3. Excellence Research Grant (ERG): Five million NPR
4. Innovative Research Grant (IRG): Ten million NPR
5. In 2022/23, 33 projects were awarded in these categories.

**Directorate of Research, Development & Innovation, Kathmandu University**

It provides funding of up to 200,000.00 NPR to its fulltime faculty members.^26^

**Dhulikhel Hospital, Research and Development Division**

It helps the faculty and staff in research design, proposal development, manuscript writing, and publication.^26^ The mechanism and amount of funding are not specified.

**Pokhara University Research Center**

This center provides funding to the faculty members. Proposal evaluation is based on independent review and previous publications. The amount provided ranges from 1,00,000 up to 3,00,000 NPR.^28^

**Maharajgunj Medical Campus under IOM**

A collaborative grant between two departments is stated to be of 100,000.00 NPR and a departmental grant of 50000.00 NPR (internal notice of the campus) in 2024.^30^ A blinded review process is applied for evaluation.

## NEPAS GRANT

NEPAS grant by Nepal Pediatric Society provides funding for research in the area of child health. The amount of funds provided is as follows: 100,000.00 NPR for life members, 500000.00 NPR for post-graduate students, medical officers, 25000.00 for post-graduate students, medical officers, and interns, and 20000.00 for nursing students.^31^

### Birat Nepal Medical Trust

This trust conducts research on infectious diseases, mental and adolescent health, and health system strengthening in Nepal. However, the amount of funds provided is not known.^32^

### Institute for Implementation Science and Health

This institute helps strengthen the capacity of implementation science research related to public health in Nepal targeting early career researchers.^34^ Only Nepali citizens can apply. The funding includes three awards of up to 100,000.00 NPR each for early career investigators and four awards of up to 50,000.00 NPR each for current graduate students.^34^

### National Institute of Health

This US institute provides grants every fiscal year to national and foreign organizations through a grant application and submission.^42^ From Nepal, Dhulikhel Hospital was awarded a total of $850,431 for two different projects from 2017 to 2021, and Kathmandu Medical College was awarded $47,772 for one project in 2019.^35^ In March 2024, the five-year grant, called Scaling Up Community-based Noncommunicable Disease Research into Practice in Pokhara Metropolitan City of Nepal (SCALE-NCD), worth $3 million was awarded.^43,44^

### Research Council of Norway

Norwegian support to research funded by Norad (Norwegian Agency for Development Cooperation) and channelled through two main partners, the Norwegian Centre for International Cooperation in Higher Education (SIU), and the RCN. RCN has 31 projects, eight of them health related. Three projects of NOK 36.0 million are solely in partnership with Nepal. The remaining projects are in partnership with other countries with funding of NOK 58.1 million.^45^

### World Health Organization

Several research projects have been funded by WHO in Nepal including the "Strengthening Pandemic Preparedness for Early Detection (SPEED)" and another project for improving the management and interoperability of health workforce data in Nepal using IntraHealth's iHRIS software.^33^

**John Hopkins University**

Johns Hopkins University funds several projects in Nepal including the first randomized controlled trialon Vitamin A supplementation among children in 1991.^46^ Typically financial support is provided for logistics, training, and implementation to ensure their success in improving healthcare outcomes in Nepal.

### C. Trend of Funding in Research

Scientific research and publications in Nepal have seen a huge increase during the last two decades. There was a more than seven-fold increase in the number of published health-related articles between 2000 and 2018.^12^ Nepal Journals Online (NepJOL), an online portal as of 2024 has 68 journals related to health care only.^47^ The research budget of UGC increased six times from 2016 to 2021.^48^

**Figure 2:**
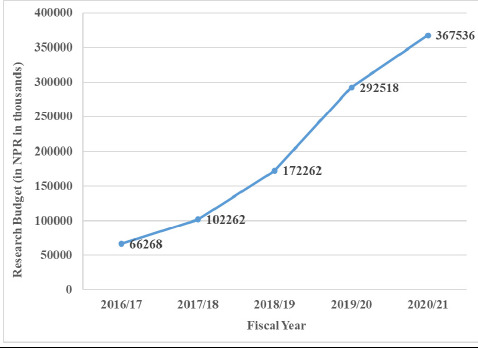
Line graph showing the incremental growth of the research budget from 2016 to 2021 (figures in Nepalese rupees in thousands).

### D. Barriers and Facilitators of Funding

In Nepal, barriers are more common than facilitators. Generally, perceived barriers are: fewer incentives for research, a less supportive environment, lack of trained manpower, lack of protected time, and a weak research culture.^2,13,19,23^ Medical student research is still in its infancy as research is not typically part of the curriculum.^16^ None of the articles describe the facilitators for funding in Nepal.

### Five key points emerged for this study:

There is a reasonably good number of funding organizations for healthcare research.

The majority of organizations have the mechanisms of funding. This has a huge implication for prospective researchers as they can better plan the scope of their research based on the expected funding amount.

There is an opportunity to increase international collaboration. Our review highlights the available international funders providing a roadmap for Nepalese researchers to tap into these resources.

Ample opportunity exists for early-stage researchers (students pursuing Masters, MPhil, or PhD degrees) to obtain funding as part of their university curriculum.

There has been an increasing growth in research and publication from Nepal, driven by the opening up of many medical colleges in the early 2000s and increased funding availability. Events, like the mega earthquake of 2015 and the recent COVID-19 pandemic have furtherprovidedopportunities for additional funding and international collaboration.

## DISCUSSION

Similar to other health metrics, Nepal is undergoing a fundamental transformation, and funding opportunities for research are no exception. This is the first scoping review from Nepal specifically looking at the funding mechanisms for healthcare research. The results further highlight the opportunities and challenges health researchers currently encounter in Nepal. Studies looking at the opportunities were predominant whereas literature on the mechanism and the potential role of the researchers were conspicuously absent.

Many researchers remain unaware of the potential availability of research funds. There has been a noticeable increase in funding in recent years, both in number and amount.^12^ Currently, at least 18 bodies provide funding for health research in Nepal-14 national and four international. The largest national funder is UGC whereas the largest international funder is RCN.

There are only a few studies performed on similar topic both in High Income Countries (HICs) and LMICs. Dandona and collegues in their review in 2017 examined the trends of funding for health research since 2001 in India.^49^ The total health research funding in 2011-12 was US$ 1.42 billion with an annual increase of 8.8%. Of this, 95% funding came from India that included 79% by the pharmaceutical industry. A study performed in Taiwan in 2020 showed an increasing trend in research and publication output and an steady rise in funded studies.^50^ Another study by Dakhil et al specifically looked at the research opportunities and funding in LMICs.^51^ They highlighted the disparity of funding opportunities for researchers between HICs and LMICs; those from LMICs mostly bearing the brunt. In their opinion, this is partly due to the lack of research infrastructure and expertise. Another relevant study by Maher et al put forth the similar disparity of research opportunities in HICs and LMICs.^52^ They discussed the ways to ensure the research equity such as the need for evidence-based decision making, and the implementation of the funding mechanism that avoids competition between excellence and equity.

Dopp et al in their paper in 2020 underscored the fragmented and limited nature of funding mechanisms for evidence based practices in behavioural health.^53^ Charani et al in 2022 highlighted significant power asymmetries in global health research funding, where most funding from HICs bypasses researchers in LMICs.^54^ They felt that the existing funding mechanisms lacked transparency and did not adequately involve LMIC researchers in decision-making processes.

**Barriers and Facilitators**

A suboptimal research environment and lack of enthusiasm are the main barriers to research. whereas none describe the facilitators in our study. Barriers are uniform across all LMICs.^4,50-54^ They include suboptimal infrastructure, inadequate education and training in grant writing and publication, suboptimal funding opportunities, language barriers to the publication and presentation of research outputs, and cost associated to travel to attend conferences.

However, based on our experience, ample availability to do small-scale research and the opportunity to conduct research on diseases endemic to our region are the facilitators for research in Nepal. Many challenges stated are also gradually improving over the years. Many institutions now offer research grants. Similarly, the number of experts and scholars interested in research careers is increasing. Multiple collaborative projects during the COVID-19 pandemic catalyzed the strengthening of the existing research capacity.^55^ To strengthen human capacity, research methodology training is being carried out all over the country. Kathmandu University in collaboration with NHRC is planning to start a Master's program in 'Research Methodology' in the year 2024.

Some solutions to the existing funding limitation are put forth by Charani et al. The need for direct funding to LMICs to build local capacity and ensure locally driven research is one such solution.^54^ Their recommendation for funders was to foster diversity in research teams, supporting capacity-building initiatives, and involving LMIC researchers in all stages of the research process. The solutions offered by Dakhil et al include performing collaborative research and streamlining research with the usual medical care.^51^

## LIMITATION

There was conspicuous absence of information regarding how health researchers in Nepal contribute to the existing funding policy. Although this is a comprehensive review originating from Nepal, we may have missed some studies, especially in the grey literature that would have answered this particular question.

## CONCLUSIONS

This review reflects the current status of research funding in Nepal. Most studies focused on the opportunities available along with the barriers to conducting research but lacked a description of the mechanism. There are mechanisms and funders in place; some are well-known and provide large amounts while others are emerging and their support is symbolic.
